# Analysis of the effect of the pre-examination and triage system in patients with fever in the fever outpatient clinic

**DOI:** 10.3389/fmed.2026.1689990

**Published:** 2026-03-09

**Authors:** Ying Li, Yafen Liu, Ying Zhou, Xiaofeng Xu, Yi Li, Wenyue Zhang

**Affiliations:** 1 Department of Infectious Disease, Peking University People's Hospital, Beijing, China; 2 Department of Emergency, Peking University People's Hospital, Beijing, China

**Keywords:** hyperpyrexia, medical service, outpatient clinics, pre-examination, triage

## Abstract

**Objective:**

To evaluate the effect of a pre-examination and triage system on patient treatment efficiency and medical staff satisfaction and to explore its application value in clinical practice.

**Method:**

This study employed a historical controlled trial design. A total of 120 patients with fever who visited our fever outpatient clinic between April and May 2023 were recruited as the control group using the convenience sampling method. One hundred twenty patients with fever who visited the clinic between April and May 2024 were recruited as the study group via the same method. In the study group, the pre-examination and triage system was used to pre-examine and triage the patients. The triage time, waiting time, incidence of febrile seizures, average number of hospitalization days, readmission rate and satisfaction of patients and medical staff were compared between the two groups.

**Results:**

The average triage time of the study group was 10.25 ± 4.58 min, which was significantly lower than the 15.66 ± 4.62 min of the control group (*p* < 0.001). The waiting time was reduced from 46.05 ± 9.79 min in the control group to 30.49 ± 9.07 min (*p* < 0.001) in the study group. The satisfaction of patients and medical staff was significantly improved, indicating that the pre-examination and triage system effectively enhanced the treatment experience and job satisfaction.

**Conclusion:**

The implementation of the pre-examination and triage system significantly improved treatment efficiency and patient satisfaction as well as the work experience of medical staff, meaning the system has practical promotion value.

## 1 Introduction

In the modern medical system, as a medical facility specializing in the treatment of acute symptoms such as fever, the fever outpatient clinic bears important public health responsibilities (1). The pressure on fever outpatient clinics has substantially increased recently, especially in the context of frequent outbreaks of infectious diseases, such as influenza, pneumonia and the recent coronavirus pandemic. Consequently, patients' medical experience and the work efficiency of medical staff have been affected (2). Patients in fever outpatient clinics typically present with a fever as the primary symptom, encompassing a range of possible infectious and non-infectious diseases (3). At the peak of the pandemic, the number of patients soared. The traditional medical model is registration, followed by triage and then a physician visit; however, the triage process is not adjusted according to the severity of the patient's condition, often making it difficult to meet rapid and efficient medical needs. According to one survey, the average waiting time of patients in fever outpatient clinics during peak hours can be several hours in some hospitals (4). This not only results in patient dissatisfaction but also increases the workload of medical staff and may even lead to a series of medical emergencies, such as febrile seizures or convulsions (5, 6). This situation poses a major risk to the medical safety of certain populations, including pregnant women, children and elderly patients, and also presents risks to the surrounding environment and public health of the hospital (7). Therefore, how to effectively organize and manage the flow of patients in fever outpatient clinics and improve the quality of medical services has become an important issue in current medical management. To address this situation, a pre-examination and triage system has gradually been introduced as a new medical management model (8). Through a preliminary assessment of the patient's symptoms, the model classifies patients according to the severity of the disease to achieve rapid and reasonable patient flow (9). Following this system, severely affected patients can obtain medical services quickly, whereas patients with relatively mild conditions undergo corresponding observation or treatment procedures, thereby reducing the pressure on the fever outpatient clinic. The implementation of the pre-examination and triage system improves the efficiency of patients' visits and reduces unnecessary waiting time. Furthermore, reasonable triage helps reduce the risk of cross-infection and ensures the medical safety of patients (10, 11). The role of this model is especially important in the case of an infectious disease pandemic, as it helps to ensure the operational order of hospitals and reduce the risk of infection in medical staff (12).

In recent years, the volume of research on the pre-examination and triage model has gradually increased at home and abroad. Relevant literature shows that the application of this model in the emergency and other departments has demonstrated good results. For example, several studies have shown that triage in the emergency department can notably improve the speed of treatment of critically ill patients and reduce the waiting time in the hospital (13). In a specific study of a fever outpatient clinic, following the implementation of hierarchical management, the utilization rate of medical resources for patients was significantly improved and satisfaction was increased (14). However, systematic research on patients with fever remains scarce, and there is a lack of in-depth analysis and summary of the specific application effects.

The innovation of the present study lies in the combination of outlining the theoretical framework of the pre-examination and triage system, conducting in-depth analysis of its specific application effect in the management of patients with fever and addressing the research gap in related fields. By comparing patient data from different periods, the study evaluates the effect of the management model in actual operation from multiple dimensions, providing a reference for subsequent clinical practice. In addition, the study assesses the satisfaction of patients and medical staff to better understand the impact of the pre-examination and triage system on hospital service quality.

Specifically, this study evaluates whether a pre-examination and triage system can significantly shorten triage time, waiting time and length of hospital stay, reduce the incidence of febrile seizures and readmission rate, and improve patient and medical staff satisfaction in fever outpatient departments. We aim to improve the management level of fever outpatient clinics, provide a theoretical basis for the promotion of similar models in the future and contribute to the reform and development of the public health system in China.

## 2 Study participants and methods

### 2.1 Study participants

In this clinical trial study, the mean triage time was adopted as the main evaluation indicator. According to the preliminary experiment, the estimated parameters of the study group and the control group were 12.25 ± 4.48 and 15.16 ± 4.41, respectively. The group population ratio of the study group and control group was 1:1. When α = 0.05 (two-sided test) and power (1–β) = 90%, the calculated sample size of each group was 92. Considering a 10% dropout factor, 101 cases were required for each group, with a total of 202 cases. The sample size calculation can be expressed as follows:


$$\begin{array}{l} n=\frac{{\left(z_{1-\alpha /2}+Z_{1-\beta }\right)}^{2}•\left(\sigma_{c}^{2}+\sigma_{h}^{2}\right)}{{\left(\mu_{c}-\mu_{h}\right)}^{2}} \end{array}$$ (1)

This study was a prospective cohort study with a historical contemporaneous control. Given the workforce, material resources and time required for the study, a convenience sampling method was used, and patients with fever who were treated in the Peking University People's Hospital's fever outpatient clinic were selected as the research participants. The participants were divided into two groups. The control group comprised 120 patients with fever who visited the fever outpatient clinic of the hospital between April and May 2023. This group of patients did not use the pre-examination and triage system and underwent the conventional treatment process. In the study group, 120 patients with fever who visited the fever outpatient clinic of the hospital between April and May 2024 were selected. This group of patients was managed according to the pre-examination and triage system to compare the effects before and after the intervention. The general data of the patients were collected, including age, gender and average fever temperature.

The inclusion criteria were as follows: (1) adults aged >18 years; (2) patients presenting with body temperature ≥37.5 °C; (3) patients who were willing to participate in the study, signed informed consent forms and had a full understanding of the purpose of the study and related procedures; and (4) patients initially clinically diagnosed by routine assessment methods and suitable for pre-examination and triage management (an initial assessment may have been performed and then graded according to the patient's clinical presentation, history and signs).

The exclusion criteria included (1) patients with acute or severe underlying diseases, such as severe cardiopulmonary disease or renal insufficiency; (2) patients with a history of mental illness or current mental instability; (3) patients or their families who refused or did not cooperate with the diagnosis and treatment plan and research purpose; and (4) pregnant and lactating women.

### 2.2 Fever clinic service process and triage system

#### 2.2.1 Basic service process of fever clinic

1. Service time: The clinic operates 24 h a day, with dedicated staff present during peak hours (08:00–22:00). The daily patient volume prior to intervention (2023) was 80–120 cases per day, with seasonal peaks (e.g. flu season) exceeding 150 cases per day. Staffing includes 8–12 physicians (including three infectious disease specialists on rotating shifts) and 15 nurses (with four dedicated triage positions post-intervention).
2. Collaboration mechanism: Patients identified during triage as having conditions such as sepsis or respiratory failure are immediately transferred to the emergency department for urgent care. The emergency department provides nighttime imaging services (computed tomography/magnetic resonance imaging) and laboratory tests. Joint treatment plans are developed for suspected severe infections.

#### 2.2.2 Conventional process of control group (2023)

1. Registration: Patients register at the front desk.
2. Basic triage: Nurses measure body temperature and heart rate without conducting standardized severity assessments.
3. Queue rules: All patients are placed in a single queue based on registration order, regardless of the severity of their condition.
4. Reception principle: Physicians see patients in the order of registration, with priority given only to those exhibiting critical symptoms (e.g. shock, major bleeding).

#### 2.2.3 Implementation method of pre-triage and triage system (2024)

The implementation of the pre-triage and triage system involves the following steps:

1. Establishment of grading standards: Based on clinical manifestations, medical history and physical signs, clear grading standards are established in advance. Patients are categorized into three levels: severe, requiring immediate treatment (e.g. unstable vital signs, acute massive haemoptysis or gastrointestinal bleeding, shock accompanied by breathing difficulties, and consciousness disorders); moderate, requiring rapid assessment but not emergency treatment (e.g. fever accompanied by other symptoms, such as cough or muscle pain); and mild, suitable for routine examination and observation (e.g. fever with no obvious accompanying symptoms).
2. Training of Medical Staff: Medical personnel involved in pre-triage and triage are trained so they become familiarized with the grading standards and treatment protocols, ensuring adherence to unified guiding principles during implementation.
3. Implementation of four-level screening in pre-examination and triage of fever clinics: At the security post at the hospital entrance, two guidance staff are assigned to measure body temperatures. These staff members will, at the first opportunity, instruct patients with fever and accompanying family members on how to wear disposable medical masks correctly and guide them to the pre-examination and triage desk.

Nurses at the pre-examination and triage desk conduct the second-level pre-examination and triage screening. In accordance with the hospital's formulated pre-examination and triage procedures, the nurses conduct rapid and accurate condition assessments for patients seeking medical treatment. Based on the different conditions of patients, they will be promptly directed to the fever clinic, isolation observation area or intensive care unit negative pressure isolation ward according to the designated route plan.

Nurses in the fever clinic perform the third-level triage screening. With strict adherence to the principle of “one doctor, one patient, one consulting room,” after the attending nurse completes a re-assessment, the treatment order is arranged in a reasonable manner based on the severity of the patient's condition and infection risk level, with the patients and their family members comforted in a timely manner. Another nurse will collect throat swab samples to improve the efficiency of the diagnosis and triage.

Physicians attending the fever clinic conduct the fourth-level diagnosis and screening. The physicians conduct systematic and comprehensive medical examinations and assessments, perform accurate triage and referrals and ensure the safety of patients seeking medical treatment. Once a suspected infection case is identified, isolation, observation and treatment are immediately initiated. In addition, medical staff who have had close contact with the patient will undergo medical observation, with daily temperature measurements and infection risk screening.

### 2.3 Evaluation indicators

#### 2.3.1 Clinical indicators

In terms of clinical measures, the mean triage time difference and mean waiting time were compared between the study and control groups. The triage time was defined as the time from admission to the fever outpatient clinic to the initial examination. The waiting time was defined as the time from admission to the fever outpatient clinic to the initiation of the diagnosis and treatment of the patient by the physician. All time points were recorded by nurses. The incidence of febrile seizures in the two groups of patients was calculated to evaluate the effect of the pre-examination and triage system in reducing accidents. The number of days during hospitalization was also recorded, and the effect of the pre-examination and triage system on hospitalization time was analyzed. The readmission rate of the two groups of patients within 30 days after discharge was calculated to evaluate the impact of pre-examination and triage on the follow-up management of patients.

#### 2.3.2 Patient and medical staff satisfaction

This study used a questionnaire survey to evaluate the satisfaction of patients and medical staff with the pre-examination and triage system. The questionnaire was scored using a four-level Likert scoring method: four points = “very satisfied,” three points = “satisfied,” two points = “dissatisfied” and one point = “very dissatisfied.” For patient satisfaction, the questionnaire covered the following aspects: (1) quality of the triage, including the fluency and convenience for patients in the treatment process; (2) waiting time (to understand the patient's perception of waiting time and satisfaction); (3) the order of waiting, which encompassed the patient's evaluation of the waiting environment and order; (4) satisfaction with medical treatment priority based on hierarchical management; and (5) service satisfaction, which involved a comprehensive assessment of patient satisfaction with the entire treatment experience. While patients waited in the fever clinic, questionnaires were distributed and completed with the help of nurses with unified training. The patients' details were recorded and the questionnaires were handed over to the nurse for preservation, with those of pediatric patients completed by accompanying family members. If other patients could not complete the questionnaire themselves, accompanying family members would help them. One hundred twenty questionnaires were distributed among the patients in the control and study groups, and the response rate was 100%.

For the satisfaction survey of medical staff, the contents included the following: (1) convenience of use (to evaluate the ease of use of the pre-examination and triage system for medical staff); (2) order of waiting (to grasp the medical staff's perception of the order of waiting for patients); (3) priority of treatment (the satisfaction of medical staff regarding the priority management of patients); triage accuracy (medical staff's evaluation of the accuracy of the triage system); and (4) patient compliance (to investigate the satisfaction of medical staff regarding patients following the doctor's advice; Supplementary material).

The medical staff satisfaction questionnaire was aimed at a total of 25 personnel, including doctors and graded pre-examination and rescue nurses in the fever outpatient clinic. The satisfaction survey was conducted before and after the implementation of the pre-examination and triage system. Before and after the implementation of pre-screening and triage, the medical staff were the same group. At the end of shifts, nurses who had undergone unified training issued and collected questionnaires on medical satisfaction. Before the implementation of the pre-examination and triage system, a total of 25 questionnaires were issued and 25 were recovered, with an effective response rate of 100%. Following the implementation of the pre-examination and triage system, a total of 25 copies were issued and 25 were recovered, with an effective response rate of 100% (Supplementary material).

### 2.4 Statistical analysis methods

The SPSS 26.0 (IBM, Armonk, NY, USA) software package was used for statistical analysis. A normality test was performed on continuous numerical variables, and those conforming to a normal distribution were expressed as mean ± standard deviation. The two independent samples *t*-test was used for inter-group comparisons. The classification data were expressed as frequency (percentage), and the two groups were compared using the chi-square (χ^2^) test. A *p*-value < 0.05 indicated a statistically significant difference.

## 3 Results

### 3.1 Comparison of general data between the two groups of patients

In the control group, there were 66 men and 54 women, with an average age of 37.51 ± 7.58 years. In the study group, there were 61 men and 59 women, with an average age of 38.54 ± 9.94 years. The average fever temperature of the control group was 38.95 ± 0.78 °C, and that of the study group was 39.12 ± 0.88 °C. There was no significant difference in age, gender distribution or mean fever temperature between the two groups, which provided a basis for the credibility of the follow-up results, as shown in Table 1.

**Table 1:** Comparison of general data of patients [*X* ± S]/[*n* (%)].

**Variable**	**Control group (*n* = 120)**	**Study group (*n* = 120)**	***t/χ^2^***	***p*-Value**
Age	37.51 ± 7.58	38.54 ± 9.94	−0.905	0.366
**Gender**				
Male	66 (55.00)	61 (50.83)	0.418	0.518
Female	54 (45.00)	59 (49.17)		
Average heating temperature (°C)	38.95 ± 0.78	39.12 ± 0.88	−1.541	0.125
**Grading**				
Severe	21	18	1.478	0.478
Moderate	60	54		
Mild	39	48		

*p* < 0.05 indicates a statistically significant difference.

### 3.2 Comparison of clinical data between the two groups of patients following treatment

Following the implementation of the pre-examination and triage system, the clinical data of the two groups of patients were compared and analyzed. The average triage time of the control group was 15.66 ± 4.62 min, whereas that of the study group was reduced to 10.25 ± 4.58 min. The difference was significant (*t* = 9.104, *p* < 0.001), indicating that the implementation of the system significantly improved the triage efficiency. The average waiting time of the control group was 46.05 ± 9.79 min, and that of the study group was 30.49 ± 9.07 min, which also indicated a significant time reduction (*t* = 12.766, *p* < 0.001). The incidence of febrile seizures was 10.83% in the control group and 8.33% in the study group. The statistical analysis of this index showed no significant difference. The average number of hospitalization days of the control group was 6.82 ± 2.22 days, and that of the study group was 6.35 ± 1.91 days. There was no significant statistical difference (*t* = 1.745, *p* = 0.082). The readmission rate was 13.33% in the control group and 10.83% in the study group, and no significant difference was found (χ^2^ = 0.353, *p* = 0.552). The above results indicate that the pre-examination and triage system demonstrated an outstanding performance in reducing triage and waiting time, whereas there was no significant difference in the other indicators (see Table 2).

**Table 2:** Comparison of clinical data before and after the implementation of the pre-examination and triage system [*X* ± S]/[*n* (%)].

**Variable**	**Control group (*n* = 120)**	**Study group (*n* = 120)**	***t/χ^2^***	***p*-Value**
Average triage time (minutes)	15.66 ± 4.62	10.25 ± 4.58	9.104	<0.001
Average waiting time (minutes)	46.05 ± 9.79	30.49 ± 9.07	12.766	<0.001
Incidence of febrile seizures convulsion (%)	13 (10.83)	10 (8.33)	0.433	0.511
Average hospitalization time (day)	6.82 ± 2.22	6.35 ± 1.91	1.745	0.082
Readmission rate (%)	16 (13.33)	13 (10.83)	0.353	0.552

*p* < 0.05 indicates a statistically significant difference.

### 3.3 Comparison of patient and medical staff satisfaction between the two groups following treatment

#### 3.3.1 Comparison of patient satisfaction

In terms of triage quality, the control group scored 1.98 ± 0.53, and the study group scored 3.51 ± 0.52, which indicated a significant improvement (*t* = −22.017, *p* < 0.001). Regarding waiting time, the score of the control group was 1.94 ± 0.27, and that of the study group was 2.96 ± 0.35, which also indicated a significant improvement (*t* = −25.124, *p* < 0.001). In terms of waiting order, the control group scored 2.13 ± 0.43, and the study group scored 3.01 ± 0.09. Statistical analysis showed a significant difference (*t* = −21.870, *p* < 0.001). Regarding treatment priority, the score of the control group was 1.83 ± 0.38, and that of the study group was 3.81 ± 0.40, with a significant difference (*t* = −39.547, *p* < 0.001). For service satisfaction, the control group scored 2.38 ± 0.49, and the study group score increased to 3.64 ± 0.48, with a significant difference (*t* = −20.101, *p* < 0.001). These results demonstrate that patients' satisfaction with the pre-examination and triage system was significantly improved in all dimensions (see Table 3).

**Table 3:** Comparison of patient satisfaction scores after the implementation of the pre-examination and triage system [*X* ± S].

**Group**	**Triage quality**	**Waiting time**	**Waiting orderliness**	**Priority of treatment**	**Service satisfaction**
Control group	1.98 ± 0.53	1.94 ± 0.27	2.13 ± 0.43	1.83 ± 0.38	2.38 ± 0.49
Study group	3.51 ± 0.52	2.96 ± 0.35	3.01 ± 0.09	3.81 ± 0.40	3.64 ± 0.48
*t*	−22.742	−25.124	−21.870	−39.547	−20.101
*p*-Value	<0.001	<0.001	<0.001	<0.001	<0.001

*p* < 0.05 indicates a statistically significant difference.

#### 3.3.2 Comparison of medical staff satisfaction

In terms of ease of use, the control group scored 2.19 ± 0.40, and the study group scored 3.48 ± 0.50, which indicated a significant improvement (*t* = −22.017, *p* < 0.001). Regarding waiting order, the control group score was 2.09 ± 0.32, and the study group score was 3.38 ± 0.49, and the difference was significant (*t* = −24.213, *p* < 0.001). In terms of treatment priority, the control group scored 2.11 ± 0.31, and the study group 3.95 ± 0.22, with a significant difference (*t* = −52.924, *p* < 0.001). For triage accuracy, the control group score was 2.87 ± 0.34, and the study group score was 3.67 ± 0.47, indicating a significant improvement (*t* = −15.016, *p* < 0.001). Regarding patient compliance, the control group scored 1.91 ± 0.34, and the study group scored 3.81 ± 0.40, which indicated a significant improvement (*t* = −39.776, *p* < 0.001). The above results demonstrate that the implementation of the pre-examination and triage system significantly enhanced the satisfaction of medical staff, indicating the effectiveness of the system in optimizing the treatment process and improving the quality of service (see Table 4).

**Table 4:** Comparison of satisfaction scores of medical staff after the implementation of pre-examination and triage system [*X* ± S].

**Group**	**Ease of use**	**Waiting order**	**Treatment priority**	**Triage accuracy**	**Patient compliance**
Control group	2.19 ± 0.40	2.09 ± 0.32	2.11 ± 0.31	2.87 ± 0.34	1.91 ± 0.34
Study group	3.48 ± 0.50	3.38 ± 0.49	3.95 ± 0.22	3.67 ± 0.47	3.81 ± 0.40
*t*	−22.017	−24.213	−52.924	−15.016	−39.776
*p*-Value	<0.001	<0.001	<0.001	<0.001	<0.001

*p* < 0.05 indicates a statistically significant difference.

## 4 Discussion

After implementing the pre-examination and triage system, the triage time and waiting time of patients were significantly reduced, and the satisfaction of patients and medical staff was also improved. This indicates that the pre-examination and triage system enables effectively optimizing medical processes, improving system efficiency and rationally allocating resources, thereby improving the medical experience of patients. At the same time, the burden and work pressure of medical staff can be reduced and the work efficiency improved. The research shows that the pre-examination and triage system involves the preliminary evaluation of the patient's condition, which is divided into different triage priorities to ensure rational allocation of medical resources. The system has been implemented in many countries and regions and has achieved good results. Scientific and rational grading can effectively shorten patient waiting times, reduce unnecessary waste of medical resources and improve patient satisfaction (15–18). The results of this study and previous studies suggest that this system can be used in patients with fever in fever outpatient clinics to improve the quality of medical care.

This study demonstrated that the implementation of the pre-examination and triage system significantly reduced the patient's triage time and waiting time. Specifically, the triage time of the study group was 10.25 ± 4.58 min, which was 5.41 min less than that of the control group (15.66 ± 4.62 min; *p* < 0.001). The waiting time was reduced from 46.05 ± 9.79 min in the control group to 30.49 ± 9.07 min in the study group, and the difference was significant (*p* < 0.001). This result indicates that the pre-examination and triage system played a positive role in improving the efficiency of patients' treatment. The results of this study are consistent with earlier findings. Kaigui et al. (19) applied an intelligent emergency pre-examination triage system in an emergency department, and the results showed that the system helped improve the triage accuracy and reduce the waiting time of patients. Another study (20) applied graded triage nursing in the pre-examination triage of patients with acute myocardial infarction, and the results showed that this approach could effectively reduce the treatment time. Under the traditional model, all patients queue up at the same stage, and the entire process from temperature measurement to consultation to examination is conducted in series, making it prone to congestion. The hierarchical pre-examination and triage system divides the complete process into four independent nodes, with specific personnel responsible for each node, forming an assembly-line operation. The pre-procedure nodes (e.g. entrance temperature measurement and mask guidance) proceed in parallel with the core diagnosis and treatment links. While the patients complete the initial screening, the staff will simultaneously complete route planning and resource reservation.

The hierarchical system achieves accurate matching between patients and medical resources through assessment, avoiding the occupation of high-end resources by mild cases. At the same time, the hierarchical pre-examination and triage system can effectively avoid clinic suspension caused by a pandemic through risk pre-positioning and isolation control.

In terms of patient satisfaction, the results showed that the implementation of the pre-examination and triage system led to a significant increase in patient satisfaction scores in multiple dimensions. The patients' satisfaction with the quality of triage scored 3.51 ± 0.52 in the study group, which was significantly different from the score of 1.98 ± 0.53 in the control group (*p* < 0.001). Similarly, the scores for waiting time satisfaction, waiting order and other indicators were also significantly improved. This indicates that the improvement in patient experience is closely related to the improvement in treatment efficiency, and that the implementation of the system achieves good results in optimizing the medical treatment process. The satisfaction of medical staff also exhibited significant improvement, with all evaluation indicators significantly higher in the study group than in the control group (*p* < 0.001). In terms of ease of use and work efficiency, the scores of the study group reflected the notable recognition and acceptance of the new system by medical staff. This result indicates that the pre-examination and triage system not only improves the patient's experience but also reduces the work pressure of medical staff and improves the working environment.

The literature reports that the implementation of the pre-examination and triage system is usually accompanied by the optimisation of the medical process and an improvement in patient satisfaction (21). Compared with existing studies, this study conducted a more systematic quantitative analysis of patient and medical staff satisfaction and ensured the reliability of the results through rigorous statistical methods. In addition, the application of this study in specific clinical scenarios provides a reference for the promotion of similar systems in other hospitals. It is worth noting that the literature also reports that the adaptability and effectiveness of the pre-examination and triage system in different types of hospitals tends to be different (22). Therefore, it is necessary to consider the specific conditions of the hospital and the characteristics of the patient group when implementing such systems.

Although this study includes valuable results, there remain some limitations that must be addressed. First, the study samples were from a single medical center, and the number of participants was relatively small, which may affect the universality and external validity of the results. Therefore, in future studies, the sample size should be expanded to enhance the reliability and generalization of the results. Second, the evaluation of the clinical effect on patients is only conducted in the short term after the implementation of the new system, and there is a lack of long-term follow-up data, thereby limiting in-depth analysis of the long-term impact of the pre-examination and triage system. Future studies should include longer-term observations to assess the impact of the system on patient health outcomes, such as complication rates. Furthermore, this study mainly focused on quantitative time, triage efficiency and satisfaction indicators and largely neglected qualitative factors, such as the emotional experience and physiological feelings of patients during the medical treatment process. Future research should combine quantitative and qualitative methods to obtain more comprehensive evaluation results.

## 5 Conclusion

By evaluating the implementation effect of the pre-examination and triage system, this study demonstrated that the system significantly improved the treatment efficiency and patient satisfaction as well as the work experience of medical staff. The results showed that the triage and waiting times were significantly reduced, and the scores of patients and medical staff in multiple satisfaction dimensions were also significantly improved. This indicates that the pre-examination and triage system plays an important role in optimizing the allocation of medical resources and improving the quality of medical services. Future promotion and application should focus on the continuous optimisation and adaptability of the system to further improve the efficiency of medical services and meet the needs of patients to achieve a better medical experience in an increasingly complex medical environment.

## Data Availability

The original contributions presented in the study are included in the article/Supplementary material, further inquiries can be directed to the corresponding author.
