# Supplementary material for: Analysis of the effect of the pre-examination and triage system in patients with fever in the fever outpatient clinic
Source: Front Med (Lausanne). 2026 Mar 9;13:1689990. doi: 10.3389/fmed.2026.1689990 (PMC13006272; doi:10.3389/fmed.2026.1689990)
Supplement: Supplementary file 1 [file Table_1.docx]

**Patients' Satisfaction with Graded Pre-screening and Triage System**

The questionnaire includes five questions, all of which are multiple-choice questions, with four options for each question, and the scores are 1, 2 and 3 in turn. Four points. The highest score for each question is 4, and the lowest score is 1.

1. Quality of triage

○Very dissatisfied

○Dissatisfied

○Satisfied

○Very satisfied

2. Waiting time

○Very dissatisfied

○Dissatisfied

○Satisfied

○Very satisfied

3. Waiting order

○Very dissatisfied

○Dissatisfied

○Satisfied

○Very satisfied

4. Priority of medical treatment

○Very dissatisfied

○Dissatisfied

○Satisfied

○Very satisfied

5. Service satisfaction

○Very dissatisfied

○Dissatisfied

○Satisfied

○Very satisfied

**Satisfaction of medical staff with grading pre-examination and triage system**

The questionnaire includes five questions, all of which are multiple-choice questions, with four options for each question, and the scores are 1, 2 and 3 in turn. Four points. The highest score for each question is 4, and the lowest score is 1.

1. Ease of use

| ○Very dissatisfied |
| --- |
| ○Dissatisfied |
| ○Satisfied |
| ○Very satisfied |

2. Waiting order

| ○Very dissatisfied |
| --- |
| ○Dissatisfied |
| ○Satisfied |
| ○Very satisfied |

3. Priority of medical treatment

| ○Very dissatisfied |
| --- |
| ○Dissatisfied |
| ○Satisfied |
| ○Very satisfied |

4. Accuracy of triage

| ○Very dissatisfied |
| --- |
| ○Dissatisfied |
| ○Satisfied |
| ○Very satisfied |

5. Patient compliance

| ○Very dissatisfied |
| --- |
| ○Dissatisfied |
| ○Satisfied |
| ○Very satisfied |
